# Progress in automating patch clamp cellular physiology

**DOI:** 10.1177/2398212818776561

**Published:** 2018-05-17

**Authors:** Luca A. Annecchino, Simon R. Schultz

**Affiliations:** Centre for Neurotechnology and Department of Bioengineering, Imperial College London, London, UK

**Keywords:** Automated electrophysiology, neuroscience, patch clamp, robotic automation

## Abstract

Patch clamp electrophysiology has transformed research in the life sciences over the last few decades. Since their inception, automatic patch clamp platforms have evolved considerably, demonstrating the capability to address both voltage- and ligand-gated channels, and showing the potential to play a pivotal role in drug discovery and biomedical research. Unfortunately, the cell suspension assays to which early systems were limited cannot recreate biologically relevant cellular environments, or capture higher order aspects of synaptic physiology and network dynamics. In vivo patch clamp electrophysiology has the potential to yield more biologically complex information and be especially useful in reverse engineering the molecular and cellular mechanisms of single-cell and network neuronal computation, while capturing important aspects of human disease mechanisms and possible therapeutic strategies. Unfortunately, it is a difficult procedure with a steep learning curve, which has restricted dissemination of the technique. Luckily, in vivo patch clamp electrophysiology seems particularly amenable to robotic automation. In this review, we document the development of automated patch clamp technology, from early systems based on multi-well plates through to automated planar-array platforms, and modern robotic platforms capable of performing two-photon targeted whole-cell electrophysiological recordings in vivo.

## Introduction

**Figure 1.**
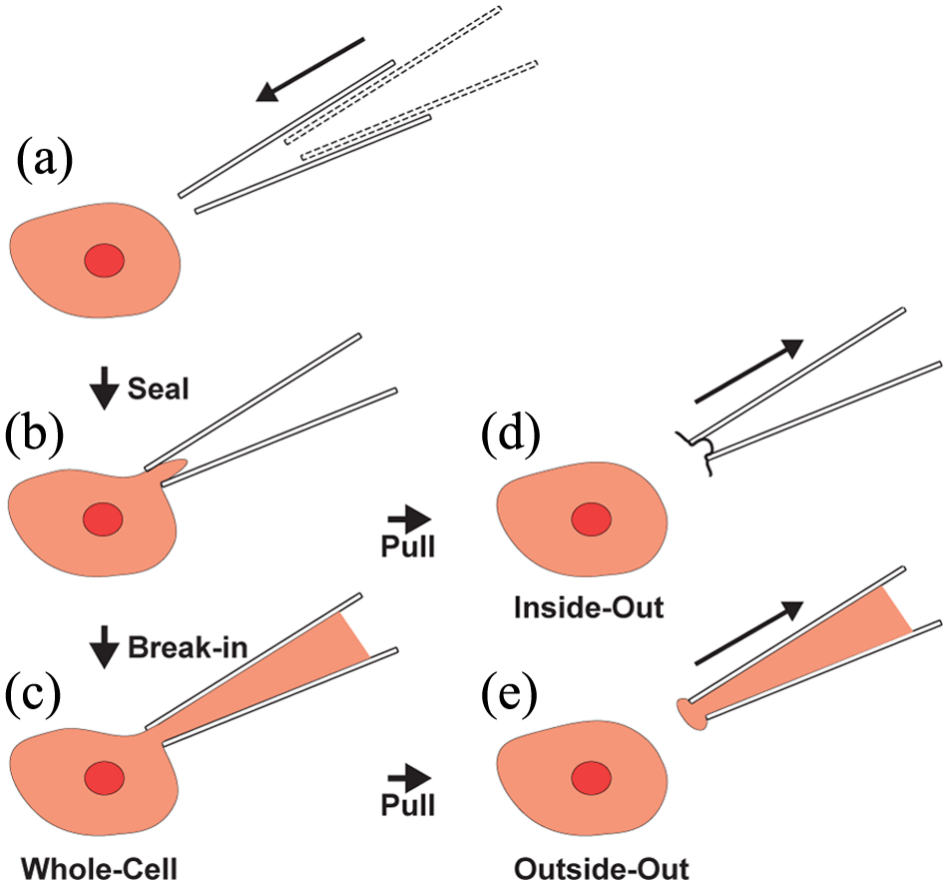
The basic patch clamp process, with variations. (a) The glass micropipette approaches the target cell. (b) Cell-attached: the micropipette is sealed onto a patch of cell membrane. (c) Whole-cell: the membrane patch is ruptured, and contact with the intracellular space is established. (d) Inside-out: if after sealing, the pipette is rapidly withdrawn, a patch of membrane remains attached to the tip, exposing the intracellular surface of the membrane to the medium. (e) Outside-out: if the pipette is gently withdrawn from a whole-cell configuration, a piece of membrane remains attached to the tip, forming a patch where the original extracellular side of the membrane remains in contact with the extracellular media, while the internal part of the membrane is in contact with the internal part of the pipette.

Patch clamp electrophysiology has transformed research in the life sciences over the last few decades, by allowing the study of ion channel biophysics, membrane properties, and synaptic responses in intact and functioning cells. The impact of this technique has been far-reaching and has been applied to a wide range of cell types, with particular relevance to excitable cells such as neurons and cardiomyocytes (Antzelevitch et al., 1991, 2007; Hamill et al., 1981; Mazzanti and DeFelice, 1990; Wellis et al., 1990). When paired with modern molecular biological techniques, the patch clamp constitutes a powerful and versatile tool in modern biology. Recording electrodes consist of glass micropipettes filled with an electrolytic solution. The basic technique (Figure 1) is performed by establishing physical contact between the electrode and the membrane of an excitable cell. Once contact is established, a slight negative pressure is applied to the pipette in order to suck a patch of the membrane into the electrode interior and attain a mechanically stable and high-impedance seal (>1 GΩ) known as a ‘gigaseal’ (Hamill et al., 1981).

Starting from the gigaseal configuration, several variations of the basic patch clamp technique can be implemented, allowing the recording and manipulation of currents flowing through single channels or across the whole-cell membrane. For the study of individual ion channels, excised patch techniques such as the inside-out (Figure 1(d)) and outside-out (Figure 1(e)) patch configurations are suitable. These configurations allow the properties of ion channels to be studied in isolation from the rest of the cell, when exposed to different chemical environments on either the intracellular or extracellular side of the patch membrane. The patch membrane can be ruptured by applying negative pressure or voltage pulses, and creating a direct contact between the cell cytoplasm and the micropipette’s internal solution. This variant is called the whole-cell recording (WCR) configuration, and it allows individual action potentials (APs) to be monitored, as well as subthreshold currents corresponding to the compound activity of multiple simultaneously conducting ion channels across the cell. Early work using the whole-cell patch clamp technique was, for practical reasons, restricted to mechanical stable in vitro preparations.

In 1991, however, the first successful in vivo WCRs of mammalian neurons were made, from cells in the cat visual cortex (Pei et al., 1991). This work was followed, sometime later, by application of the technique to the mouse (Margrie et al., 2001, 2002), which opened up the availability of additional tools from molecular biology. Compared to other animal models, mice are relatively cost-effective, easier to handle and transport, reproduce quickly, and can be inbred to yield genetically identical strains. The genetics of this animal model is relatively well understood and can be manipulated to mimic many diseases or drive specific patterns of protein expression. The development of techniques for gene targeting through homologous recombination (Doetschman et al., 1987; Thomas and Capecchi, 1987) allowed for single-nucleotide precision modification of the mouse genome and provided powerful methods for reliable genetic engineering. The generation of transgenic mouse lines expressing green fluorescent protein (GFP) in specific cells or cell classes is of particular importance, as it revolutionised the way biological structures and process are visualised in model organisms (Chalfie et al., 1994; Mombaerts et al., 1996). GFP expression is often used as a cellular indicator of successful gene manipulation (Lundstrom, 2001; Pines, 1995; Takada et al., 1997) and also allows intracellular recordings to be targeted to labelled cells in vivo using fluorescence microscopy (Komai et al., 2006a, 2006b; Margrie et al., 2003).

More than 40 years after its invention, the patch clamp paradigm remains the ‘gold standard’ approach to the study of ion channel biophysics and pharmacology, despite its inherently low throughput and manual nature (Yajuan et al., 2012). Manual patch clamp is extremely labour-intensive, requiring substantial training and innate skill of experimental personnel to successfully manipulate the pipette, control internal pressure, and seal the cell in an efficient and timely manner (DeWeese, 2007; Hamill et al., 1981). An increasing demand (especially from the pharmaceutical industry) for new tools overcoming these challenges and enabling high-throughput studies of cellular physiology has recently advanced the development of several automated electrophysiological platforms for screening of ion channel modulators (Janzen and Bernasconi 2009; Jones et al., 2009). When used to test a large number of compounds, rapidly and in parallel, using arrays of miniaturised biological assays, this paradigm is also known as high-throughput screening (HTS) (Dunlop et al., 2008; Obergrussberger et al., 2018; Py et al., 2011; Yobas, 2013; Yu et al., 2016). Automatic patch clamp systems have been implemented in vitro using planar arrays (patch-on-a-chip) with microscopic orifices for seal formation (Fertig et al., 2002; Kiss et al., 2003; Tang et al., 2010), and, to a lesser extent, glass micropipettes (Dunlop et al., 2008; Vasilyev et al., 2006). In this review, we document the development of automated patch clamp technology, from early systems based on multi-well plates through to automated planar-array platforms, and modern robotic platforms capable of performing two-photon targeted whole-cell electrophysiological recordings in vivo.

## Automated micropipette-based platforms for cellular physiology

**Figure 2.**
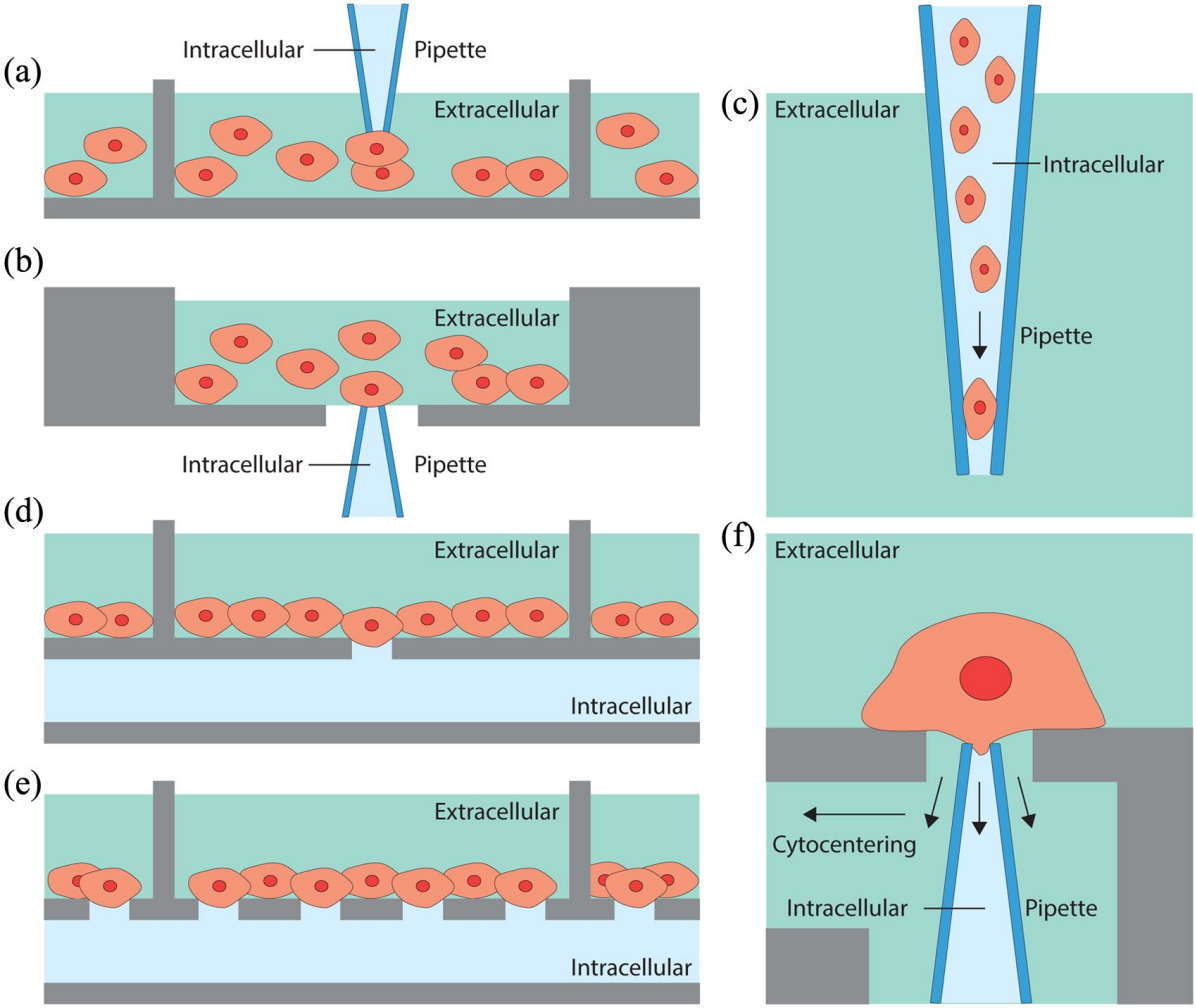
The principal automated patch clamp paradigms for studying cell suspension cultures. (a, b) The pipette moves within a cell suspension, and contacts a random cell in a layer within a density gradient (a) or at the air liquid interface (b). (c) The interior of a pipette is filled with cells in suspension, allowing the seal and whole-cell recording configuration to be achieved inside the pipette tip. (d–f) Planar-array paradigms utilise silicon or plastic-based micro-fabricated substrates allowing seal formation on a (d) single or (e) multiple micron-size orifices (population patch clamp) in wells filled with media containing cells. (f) The cytocentering paradigms consist of a pipette tip placed facing upwards at the centre of a polyimide sheet mounted at the bottom of a well which hosts suspended cells. The tip of the pipette is in the middle of an orifice in borosilicate glass surface. A cell is positioned on the recording pipette electrode using negative pressure at the suction channel; flow channel built into the chip that enables fluid exchanges. When the tip encounters a cell, suction is applied through the pipette tip to promote seal formation.

Several automated patch clamp systems based on conventional glass micropipette electrodes (Figure 2(a)–(c)) have been developed to simplify the time-consuming patching procedure and to achieve a higher throughput and reproducibility with better data quality (Asmild et al., 2003). These systems primarily used three different paradigms.

In the first approach, a pipette is moved in a density gradient solution and a cell randomly encountered in its navigation trajectory is patched either at the air/liquid interface or in a deeper cell layer (Dunlop et al., 2008; Yajuan et al., 2012). Attempts to automate this process began in the 1990s with the NeuroPatch, which then evolved as Apatchi-1 (Asmild et al., 2003), and later with the Roboocyte (ALA scientific/Multichannel Systems GmbH). These systems enabled automated cDNA/mRNA injection and subsequent two-electrode recordings in *Xenopus oocytes* on 96-well plates (Schnizler et al., 2003). More recently, Roboocyte 2 (Multichannel Systems GmbH) significantly improved the throughput of its predecessor. In the 2000s, several other systems were proposed with the promise to improve parallelisation and increase throughput: the Dynaflow (Cellectricon; now licenced to Fluicell AB), the POETs (Abbott Laboratories), a test station with six oocyte recording sites (Lehnert and Gijs, 2004) and the OpusXpress 6000 (Axon Instruments Inc., now Molecular Devices, LLC), also successfully used to rank-order α7 nicotinic receptor agonists based on functional potency and relative drug efficacy (Papke, 2006).

In the second approach, also known as cytocentering and used for mammalian cells, the pipette is placed facing upwards at the centre of a polyimide sheet mounted at the bottom of a well which hosts suspended cells (Figure 2(f)). When the tip encounters a cell, suction is applied through the pipette tip to promote seal formation (Stett et al., 2003b). The Autopatch (CeNeS Pharmaceuticals) is one such device (Dunlop et al., 2008).

The third paradigm is profoundly different to the others, as it requires no micromanipulation to obtain a stable gigaseal; rather, the interior of a pipette is filled with cells in suspension, allowing the seal and WCR configuration to be achieved inside the pipette tip (Vasilyev et al., 2005, 2006; Zhao et al., 2009). Robopatch (Axon Instruments Inc., now Molecular Devices, LLC) was one of the first systems to obtain automated ion channel recordings in inside-out configuration and was also validated for the study of ligand-gated and voltage-gated ion channels, including the voltage-gated human ether-a-go-go-related (hERG) gene product channel, and the rapidly desensitising ligand-gated α7 nicotinic receptor channel (Vasilyev et al., 2006). A similar system, but with improved throughput, is the Flyscreen 8500 robot (Flyion GmbH) which implements a modular array of independent recording pipettes (Fejtl et al., 2007; Lepple-Wienhues et al., 2003).

These systems reduce the amount of labour and attention required from the human operators, while enabling recordings comparable with manual in vitro methods in terms of quality, stability, seal formation, and resistance. Unfortunately, although providing an option for multiple recordings in parallel, the improvements in terms of throughput were relatively modest (Dunlop et al., 2008; Vasilyev et al., 2005, 2006). Parallel electrophysiological interrogation of cells can be difficult as the precise control of multiple simultaneous pipettes is a relatively challenging problem. Additional drawbacks include the need for high-quality cell suspensions and the ‘blind’ nature of the cell selection process for patching (Dunlop et al., 2008). This translates into limitations with non-uniform cell suspensions (e.g. differentiated cells derived from induced pluripotent stem cells or embryonic stem cells) and might require human intervention or entirely different paradigms. For this reason, a new planar-array-based paradigm focused heavily on automated systems for mammalian cells has emerged, challenging conventional pipette microelectrode-based approaches.

## Automated planar-array electrophysiological platforms

The planar-array paradigm utilises silicon- or plastic-based micro-fabricated substrates allowing seal formation on micron-size orifices (Dunlop et al., 2008; Py et al., 2010) (Figure 2(d) and (e)). Such systems use suspensions of cells directly pipetted into recording wells. The major advantages of this approach include increased throughput and reduced compound consumption. The first device was fabricated in silicon (Fertig et al., 2000; Hediger et al., 1999; Schmidt et al., 2000), but due to the high capacitive coupling, this was later replaced with quartz or glass (Fertig et al., 2003; Nagarah et al., 2010), polymide (Stett et al., 2003a), or polydimethylsiloxane (PDMS or silicone) (Klemic et al., 2002, 2005). Also used in lateral-patch chips with integrated fluidic channels (Chen and Folch, 2006; Ionescu-Zanetti et al., 2005; Lau et al., 2006; Tang et al., 2010), silicon allows seamless integration of multiple additional functionalities such as on-chip and amplification and processing of the bio-electrical signal (Aziz et al., 2007; Kaul et al., 2004) and has thus continued to be employed as a substrate (Matthews and Judy, 2006; Sordel et al., 2010).

Several automated electrophysiology instruments based on such technology were proposed as early as 2002: the Ion Works HT (Essen Biosciences) (Schroeder et al., 2003) and the PatchXpress (Axon Instruments Inc., now Molecular Devices, LLC) (Tao et al., 2004; Xu et al., 2003). The PatchXpress was the first planar-array-based system enabling gig-ohm seal patch clamp electrophysiology (Xu et al., 2003) and was used in support of preclinical evaluation of efficacy and safety of drug candidates targeted at cardiac ion-channels (Tao et al., 2004) and to demonstrate pharmacological modulation of the Na_*v*_1.8 (Payne et al., 2015) and Na_*v*_1.9 (Lin et al., 2016) sodium channels in pain research. The Ion Works HT was the first system to become widely available and validated as a fully automated tool for patch clamping in mammalian cells using a perforated patch clamp format (Finkel et al., 2006; Schroeder et al., 2003).

The Ion Works HT was followed by the Quattro, which introduced a population patch clamp paradigm enabling simultaneous recording of an ensemble of currents from up to 64 cells via as many as 64 orifices per well (Finkel et al., 2006). Population patch clamp significantly improved upon the success rate, compound throughput, and well-to-well variability of single-hole automated planar patch clamp (John et al., 2007). Unfortunately, electrode instability due to the much larger compound currents, the struggle to maintain continuous voltage clamp, and the inability to have the recording electrode and the pipettor heads in the well at the same time restrict the utility of this particular system, especially when dealing with voltage-gated channels.

A number of alternative platforms enabling continuous, automated voltage clamp recordings have been recently proposed: the Patchliner (Bruggemann et al., 2006), the SynchroPatch (Obergrussberger et al., 2016; Stoelzle et al., 2011) and the Port-a-Patch (all Nanion Technologies), the QPatch and Qube (Sophion Biosciences) (Asmild et al., 2003; Chambers et al., 2016; Mathes et al., 2009), several IonFLux platforms (Fluxion Bioscience) (Spencer et al., 2012), the CytoPatch (Cytocentrics) (Scheel et al., 2011), the IonWork Barracuda and FLIPR Tetra High-Throughput Cellular Screening System (Molecular Devices) (Gillie et al., 2013; Kuryshev et al., 2014), and finally, the Cellaxess Elektra platform (Cellectricon). These various instruments differ substantially in terms of patch plate design and composition, liquid handling, electrode configuration, and control software (for more details see Bell and Dallas (2017); Obergrussberger et al. (2018)).

Automated planar-array electrophysiological platforms are usually applied in the study of over-expressed ion channels in non-neuronal isolated cells lacking synaptic communication. These paradigms translate into models of questionable biological relevance as the majority of compounds targeting synaptic transmission do so by interacting directly with ion channels. In such a framework, the interrogation of multiple neuronal elements in communicating networks bears an enormous potential, but remains elusive in modern pharmaceutical assays and biomedical research (Py et al., 2011). Automated planar-array electrophysiological platforms have intrinsic limitations, in that they cannot selectively interrogate small neuronal structures (such as axons or dendrites) and generally tend to produce lower quality data in comparison to micropipette methods. For example, the relatively large leak current inherent to these platforms makes it difficult to resolve inwardly rectifying potassium channels and transient receptor channel currents (Yajuan et al., 2012). However, the limited agonist wash-out capabilities constrain data resolution and compromise the reliable and high-fidelity chemical modulation and electrophysiological interrogation of fast-desensitising ligand-gated channels. Another important consideration is the type of cell used and their viability in the experimental setting. The cell quantity required to obtain useful data tends to be relatively large, and the methodologies used to derive or acutely dissociate them and obtain homogeneous suspensions have limitations (Milligan et al., 2009).

Due to their widespread use and propensity to form high-impedance seals, Chinese hamster ovary (CHO) cells or adherent human embryonic kidney (HEK) cells are commonly used for ion channel expression in automated planar patch clamp electrophysiological platforms. Stem cells are also a viable option as demonstrated during the automatic assessment of the basic electrophysiological parameters of human induced pluripotent stem cell-derived dopaminergic neurons (Franz et al., 2017). However, different cell lines often show substantial variability with respect to the sealing and break-in process, and ad hoc protocols and optimisations are generally required during assay development.

An alternative method to study the biophysics and pharmacology of ion channels is based on self-assembled bilayer lipid membranes (BLMs). This has yielded parallel, single-channel recording enabling single-molecule analysis of membrane transporter activity (Watanabe et al., 2018), also suggesting an inhibitory effect of amyloid beta fragments on Ca^2+^-dependent K+ (hBK) channel (Kawano et al., 2013). Novel designs for improved, mechanically stable, solvent-free lipid bilayers in nano- and micro-tapered aperture have been recently proposed and used to study a cell-free synthesised cardiac potassium channel (Tadaki et al., 2017).

## Automation in brain slices

Although intrinsic to biophysical research and modern drug discovery, electrophysiological and pharmacological essays on cell suspension have limited capability to recreate biologically relevant cellular environments, let alone capturing any of the high-order aspects of synaptic physiology and network dynamics. This is especially true when considering complex pathological conditions such as neurodegenerative diseases or neuropathic pain.

The development of the living acute brain slice preparation was a pivotal achievement offering many of the advantages of an in vitro preparation while preserving several aspects of its in vivo counterparts. Extracellular field potential recordings in in vitro brain slices enable the continuous monitoring of intrinsic neuronal electrophysiological properties and their response to electrical or pharmacological stimulation. Extracellular measurements can be performed over relatively long periods of time without disrupting the cell membrane. This permits the measurement of properties such as firing rates, temporal firing patterns, and dynamic ranges and enables the categorisation of cells into functional groups that may be correlated with anatomical information in terms of spatial location, connectivity or morphology.

Automated systems for in vitro brain slice extracellular field potential recordings have been available for some years: the SliceMaster (Scientifica LTD, Merck and NPI Electronics) (Stopps et al., 2004), the Synaptic Explorer (Wyeth Neuroscience; acquired by Pfizer in 2009), Synchroslice (Lohmann Research Equipment), and the Autoslice (Lohmann Research Equipment). Automated system for brain slices recordings allowed a substantial experimental time reduction in some studies; however, they can handle only a limited range of experimental protocols (Dunlop et al., 2008). Although not automated, a different and potentially better approach for high-capacity brain-slice field recordings is to use multi-channel electrode arrays (MEAs; for a review see Obien et al. (2015)).

MEAs enable field potential recording with typical spatial resolution between 30 and 100 µm and have been extensively used to analyse network properties of neurons (Keren and Marom, 2016; Van Pelt et al., 2005; Wagenaar et al., 2005), retinas (Ishikane et al., 2005; Jacoby and Schwartz, 2017; Menzler and Zeck, 2011) and cardiomyocytes (Haraguchi et al., 2006; Sung et al., 2014; Zhu et al., 2017), and although not enabling intracellular control (and notably voltage- and current-clamp), offer potential for insights into conditions involving pathological hypo- or hyperactivity such as epilepsy. However, some recent studies attempting intracellular recordings of APs by a nanotube synthetically integrated on top of a field effect transistors have been relatively successful (Duan et al., 2012; Qing et al., 2013).

Slice field-recording paradigms can provide information about the compound activity of a population of neurons but fail to capture the contribution to the network and the intrinsic excitability of its individual components. They also cannot detect the subthreshold current that whole-cell patch clamp recordings can resolve. Whole-cell patching of multiple neurons has overcome such limitations and revealed important insights into synaptic plasticity (Perin et al., 2011). Manual in vitro patch clamp is a powerful and versatile technique, allowing the infusion of dyes and/or transfecting agents, and/or extraction of cytosolic contents for transcriptomic single-cell analysis (Eberwine et al., 1992; Qiu et al., 2012).

**Figure 3.**
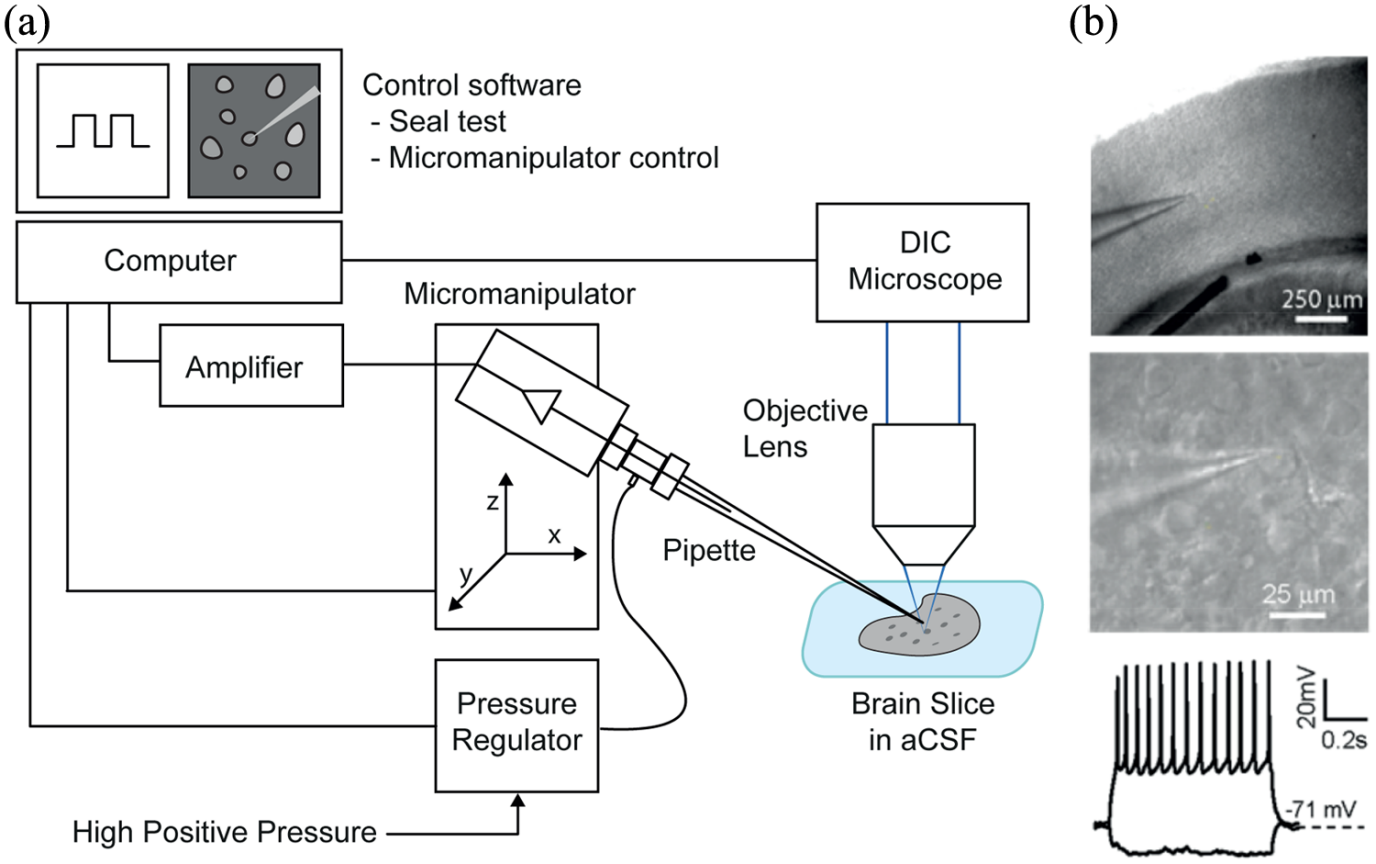
Automatic visually guided whole-cell recording in vitro. (a) Experimental apparatus. The system consists of a conventional patch setup equipped with a programmable micromanipulator, differential intensity contrast (DIC) optics with a camera, a signal amplifier, a digital/analogue acquisition board, a computer, and a custom-made electro-pneumatic actuator for controlling internal pressure of pipette. (b) Representative images show an automatically patched cell at 4× magnification (top), 40× magnification DIC optics (middle) in a mouse visual cortex brain slice, and electrophysiological responses of an automatically patch clamped neuron to hyperpolarising and depolarising current injections (bottom). Adapted from Wu et al. (2016).

A number of studies partially automating aspects of the whole-cell process have been proposed, both for in vitro (Campagnola et al., 2014; Perin and Markram, 2013) and in vivo (Long et al., 2015) patch clamp physiology. However, the first automatic image-guided (using differential interference contrast optics) patch clamp system for brain slices was presented in 2016 (Wu et al., 2016) (Figure 3). This system (which actually followed developments in in vivo automation, which will be discussed in the next section) automated all major steps of the patch clamp process in slices. Its performance was validated by performing patch clamp recordings in mouse brain slices from wild-type, transgenic, and virally injected mice. Automation of brain slice sample preparation, however, remains to be carried out.

## Automation of in vivo patching

Although the immediate cellular environment of neurons in the acute brain slice preparation is thought to remain largely intact, at least for cells well below the surface of the slice, functional long-range connections are lost. As well as a significant loss of glutamatergic synaptic input, this means a fundamentally compromised neuromodulatory environment. In addition, studies of the neural correlates of behavioural or cognitive properties must be carried out in an intact nervous system, connected to sensory inputs and motor outputs. For this reason, in vivo whole-cell patch clamp electrophysiological experiments remain crucial to progress. Indeed, in vivo WCR remains the most effective tool for high-fidelity analysis of post-synaptic responses to sensory stimuli, behavioural states, and cognitive processes in healthy and pathological conditions (Hirsch et al., 1998; Jagadeesh et al., 1997; Margrie et al., 2002). One particular advantage of this technique is that it provides a direct link between cellular and systems neuroscience approaches. Unfortunately, however, the application of WCR techniques in living animals is technically challenging. It is not unusual to have to replace the patch pipette many times during an attempt to obtain a recording, due to tip clogging or problems with internal solution (DeWeese, 2007) (tip clogging tend to be less frequent in in vitro paradigms). A mechanically stable experimental arrangement is a fundamental prerequisite for successful recordings, but is intrinsically difficult to achieve in live animal preparations, due to pulsatile motion of brain tissue. The detrimental impact of breathing- and heartbeat-related periodic brain tissue movement can be reduced but not completely removed (DeWeese, 2007; Furue et al., 2007). This type of in vivo WCR is often referred to as ‘blind’ WCR, as (unlike in vitro WCR, which typically uses differential interference contrast optics, or two-photon targeted patch (TPTP), discussed in the next section), it is performed without any form of visual targeting: essentially, the first cell encountered is recorded from, which can lead to a bias towards sampling from large, numerous cells such as pyramidal cells.

Automation of in vivo patch clamp physiology offers a number of benefits: reduction of the steep ‘learning curve’ required for new physiologists to become proficient in the technique, standardisation of recording quality, and improvement of throughput. It also opens up the potential to scale up the technique to simultaneous patch clamp recording from multiple cells in vivo, enabling assays of synaptic coupling and opening up many new questions in basic and translational neuroscience.

**Figure 4.**
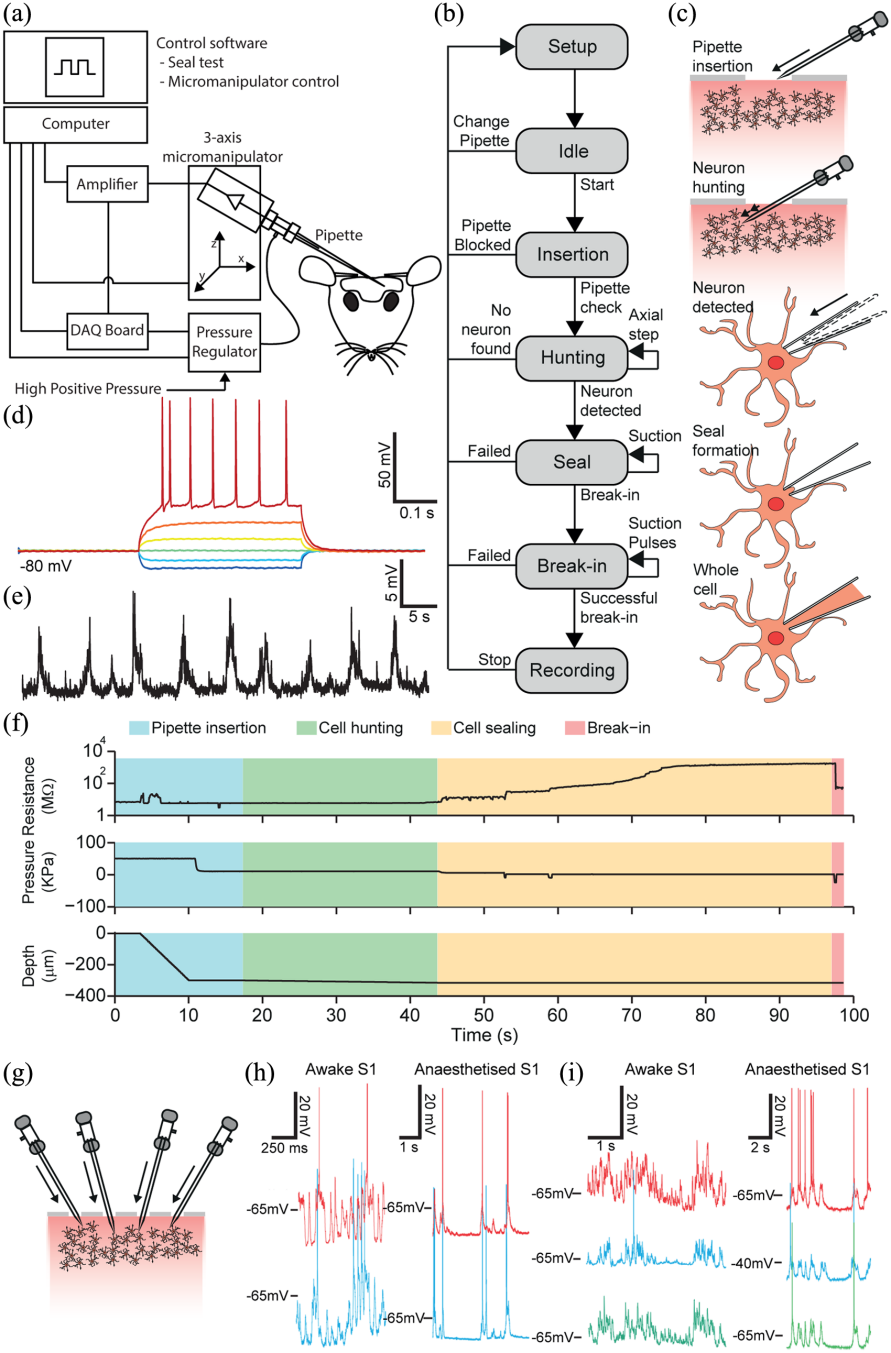
Automatic blind whole-cell recordings in vivo. (a) Experimental setup. The system consists of a conventional patch clamp rig equipped with a programmable 3-axis micromanipulator, a signal amplifier, a digital/analogue acquisition board, a computer and a custom-made electro-pneumatic actuator for controlling internal pressure of pipette. (b) Block diagram of the automated patch procedure. (c) Stages of the procedure: setup and pipette placement, insertion, cell hunting, seal formation and break-in followed by whole-cell configuration. (d) Intrinsic response of a neuron during hyperpolarising and depolarising current injection applied through the recording micropipette (400-ms-long current transients from −100 to +150 pA in 50-pA steps); pipette tip depth was 549 µm below the pial surface. (e) Current-clamp trace for the same neuron at rest. (f) Example of time-courses of pipette resistance, internal pressure and pipette depth. Adaptive suction was applied by the system to help attaining a tight seal. Cell break-in (pink region) was attained by applying one or more suction pulses. (g) Multi-patching system implemented by Kodandaramaiah et al. (2018). The system automatically guides all pipettes to the desired target region in the brain and seeks neurons. Clogged pipettes are detected and deactivated. (h) Representative voltage traces for two neurons patched simultaneously in the somatosensory cortex of awake (left) and anaesthetised (right) head restrained mouse. (i) Representative voltage traces from three neurons patched simultaneously in the somatosensory cortex of an awake (left) and anaesthetised (right) head-fixed mouse. Adapted from Annecchino et al. (2017) and Kodandaramaiah et al. (2018).

The first automatic method system for performing WCR in anaesthetised mice was developed by Kodandaramaiah et al. (2012). This system implements the principal steps involved in a manual patch clamp experiment, including micromanipulator and pipette internal pressure control, cell detection, sealing, and break-in. It detects neurons by analysing changes in electrode impedance as the micropipette is advanced through tissue and has been demonstrated to be capable of achieving performances, in terms of yields, cell recording quality, and operational speeds, comparable to human operators when tested in the mouse neocortex and hippocampus. The system achieved WCR success rates of up to 43.6% in specific tests (Kodandaramaiah et al., 2012). Annecchino et al. (2017) developed a system based on a similar approach (Figure 4(a)–(f)), achieving comparable performance (WCR success rates of up to 50%) (Annecchino et al., 2017). Both the Kodandaramaiah et al. (2012) and Annecchino et al. (2017) systems were programmed in the LabView (National Instruments) data acquisition and control software language.

Desai et al. (2015) described an alternative (MATLAB-based) implementation of an automated system for in vivo WCR, demonstrating it in awake head-fixed mice running on a wheel, obtaining a success rate of 17%. Recently, the capabilities of Kodandaramaiah et al. implementation were scaled up to allow the independent control of four micropipettes and obtain WCRs from up to three different neurons at the same time in the neocortex of both anaesthetised and wake animals (Figure 4(g)–(i)) (Kodandaramaiah et al., 2018). This system obtained a dual or a triple WCR in 18% of the trials in awake mice and 29% of trials on anaesthetised mice; at least one WCR was obtained in 90% of the cases. The system is also relatively fast: trials took on average 10.5 ± 2.6 min to yield recordings lasting up to 14.0 ± 10.0 min in the awake head-fixed mouse. This development, of systematic automated whole-cell patch clamp electrophysiology in awake behaving mice, opens up numerous important questions in systems neuroscience to the technique and may soon yield advances in our understanding of the cellular basis of perceptual, motor, and cognitive behaviour.

## Automation of two-photon targeted in vivo patch clamp physiology

Common to all the systems for in vivo patch clamping described above is that they are carried out ‘blind’. This means that the genetic and morphological identity of the recorded neuron remains unknown until post hoc histological analysis is undertaken. In practice, this largely restricts the technique to large, common neuronal cell types such as pyramidal cells, as smaller and less numerous cell types are encountered insufficiently frequently to generate sufficient sample sizes to make scientific conclusions.

The lack of selectivity of classic ‘blind’ in vivo patching approaches has been overcome by combining intracellular recording with two-photon laser scanning microscopy. Specific cell classes can be labelled by a number of methods, including creating transgenic mice, either by crossing Cre driver lines with reporter mice (Madisen et al., 2010) or otherwise (Margrie et al., 2003), or using viral vectors designed to drive expression via cell-specific promoters (Dittgen et al., 2004). In some cases, sufficient visual information may be available to identify cell types after labelling tissue with acetoxymethyl (AM) ester fluorescent indicators (Schultz et al., 2009; Stosiek et al., 2003), or ‘shadowpatching’ (ejecting dye into the tissue to reveal cell location and morphology via a negative image) (Kitamura et al., 2008).

By adaptively planning and executing image-guided pipette motion in the intact brain, it is possible to obtain recordings from specific cell, cell classes, or cell compartments (i.e. dendrites) based on morphological, genetic, or electrophysiological signatures (Komai et al., 2006a, 2006b; Margrie et al., 2003). Targeted electrophysiological interrogations allow for neuronal network-network related functional examination and the study of the physiological role of cell type–specific proteins in orchestrating neuronal responses (Komai et al., 2006a, 2006b) and when combined with either live imaging or postmortem histological assay, can provide morphological correlates of cell physiology.

Direct visualisation of seal formation can improve the success rate, recording quality, and stability, by allowing for the relative cell-electrode position to be continuously optimised, and for the seal formation to be initiated (by applying holding voltages and suction) at the optimal time (Kitamura et al., 2008; Komai et al., 2006a). Another important application enabled by two-photon targeting is in vivo targeted electroporation of dyes (Nevian and Helmchen, 2007) and DNA (Judkewitz et al., 2009; Kitamura et al., 2008) by extracellular stimulation via patch pipettes targeted at individual cells. Furthermore, by augmenting the patch pipette internal solution with activity-dependent dyes, simultaneous measurement of electrical and chemical signalling can be obtained from the same cell.

Despite the wealth of knowledge provided by TPTP, studying the roles of individual cells in live tissue remains a substantial challenge. The insertion of a pipette into soft tissue causes viscoelastic morphological deformation which, in turn, induces target migration away from its initial position with respect to a solid frame of reference. Precise vision-guided control of pipette motion and microscope objective viewpoint generation for targeted single-cell electrophysiological interrogation is an inherently challenging and highly specialised task requiring close attention and extensive expertise. The whole engagement procedure, when performed manually, is difficult, tedious, and characterised by a very low throughput. To address this issue, Long et al. (2015) presented a semi-automatic method to direct a micropipette to a user-selected cell using volumetric image data collected at an intermediate point along the route to the target. However, this strategy is not sufficiently accurate for automated targeted recordings and requires human intervention to perform the final cell engagement, seal, and recording.

**Figure 5.**
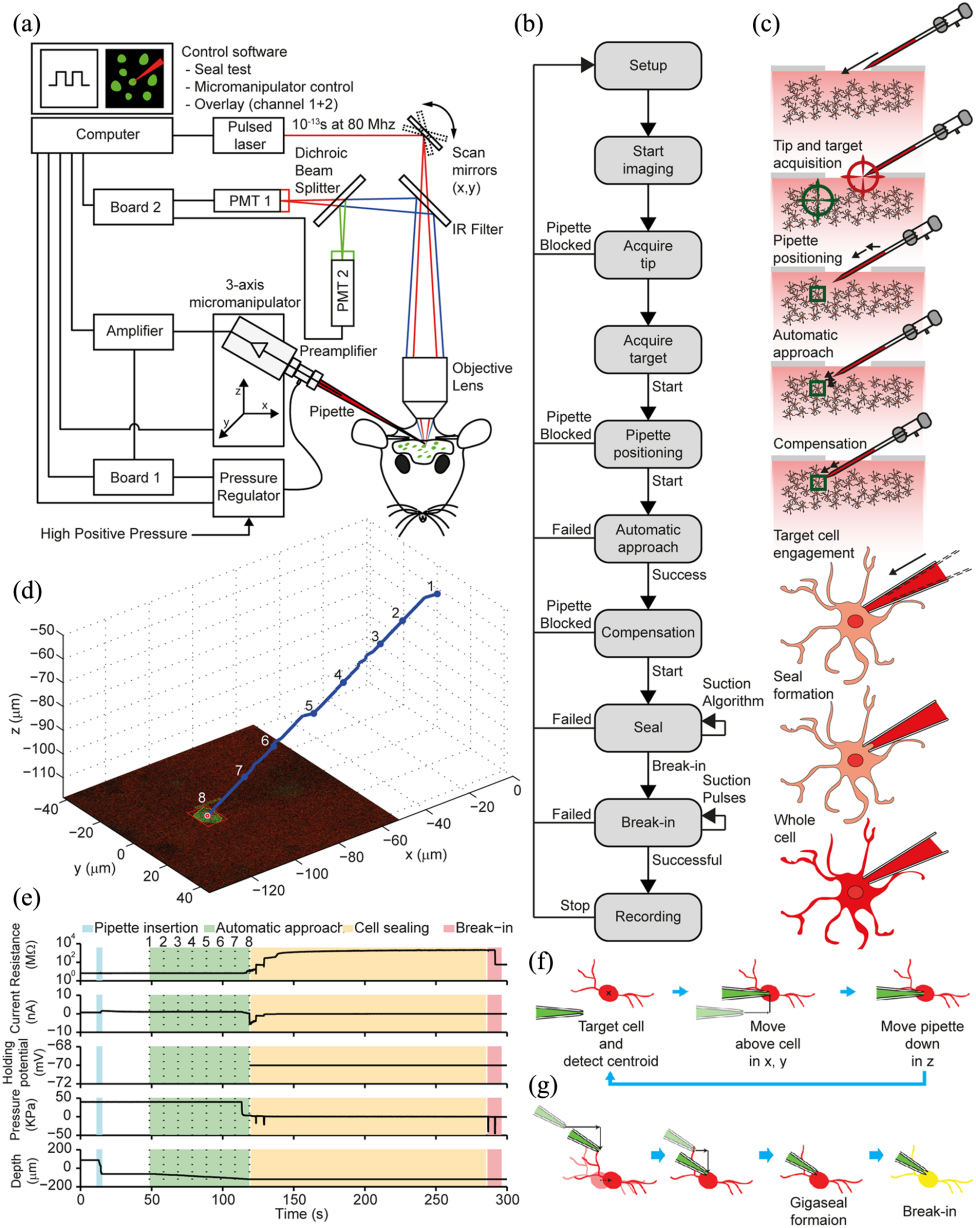
Automated two-photon guided whole-cell recording in vivo. (a) Schematic of the apparatus, which consists of a conventional commercial two-photon microscope, a mode-locked Ti-Sapphire laser, a patch setup equipped with programmable three-axis micromanipulator, a signal amplifier, an analogue to digital converter board, a computer and custom-made electro-pneumatic actuator for controlling micropipette internal pressure. (b) Block diagram of the two-photon guided robotic procedure. (c) Stages of the visually guided procedure: setup and pipette placement, tip and target coordinates acquisition, pipette positioning, automatic approach, position compensation, target cell engagement, seal formation, and break-in followed by whole-cell configuration. (d) Pipette approach trajectory and state during a typical robotic navigation towards the target cell, with real-time feedback control of trajectory enabled. (e) Time-course of pipette resistance, current, holding potential, internal pressure and depth during the patching procedure (stages colour coded; numeric labels correspond to points on the approach trajectory in (d)). (f) Schematic of the alternate navigation method for final approach implemented by Suk et al. (2017) (viewed from above). (g) Lateral view of (f) cell approach, gigaseal formation, and break-in. Adapted from Annecchino et al. (2017) and Suk et al. (2017).

Considerable advances in automating two-photon targeted WCR were recently made by Annecchino et al. (2017) and Suk et al. (2017). These authors independently reported two alternative implementations of an image-guided platform capable of tracking the light signatures labelled cell bodies, while automating the target engagement process and all the steps involved in the procedure of patch clamping specific cell types in vivo (Figure 5(a)–(e)). Both systems achieve gigaseal-tight contact between patch pipette and cellular membrane, stability, and good quality WCR configuration comparable to manually conducted experiments. One of the key technological challenges has been achieving persistent monitoring of the target–object position by an autonomous computer vision system to assess and compensate for movement of tissue induced by insertion of the pipette into the brain. The automatic fine control of the pipette position for navigation and target engagement relies on the real-time analysis of the light signature of the fluorescent targets in the intact brain.

**Figure 6.**
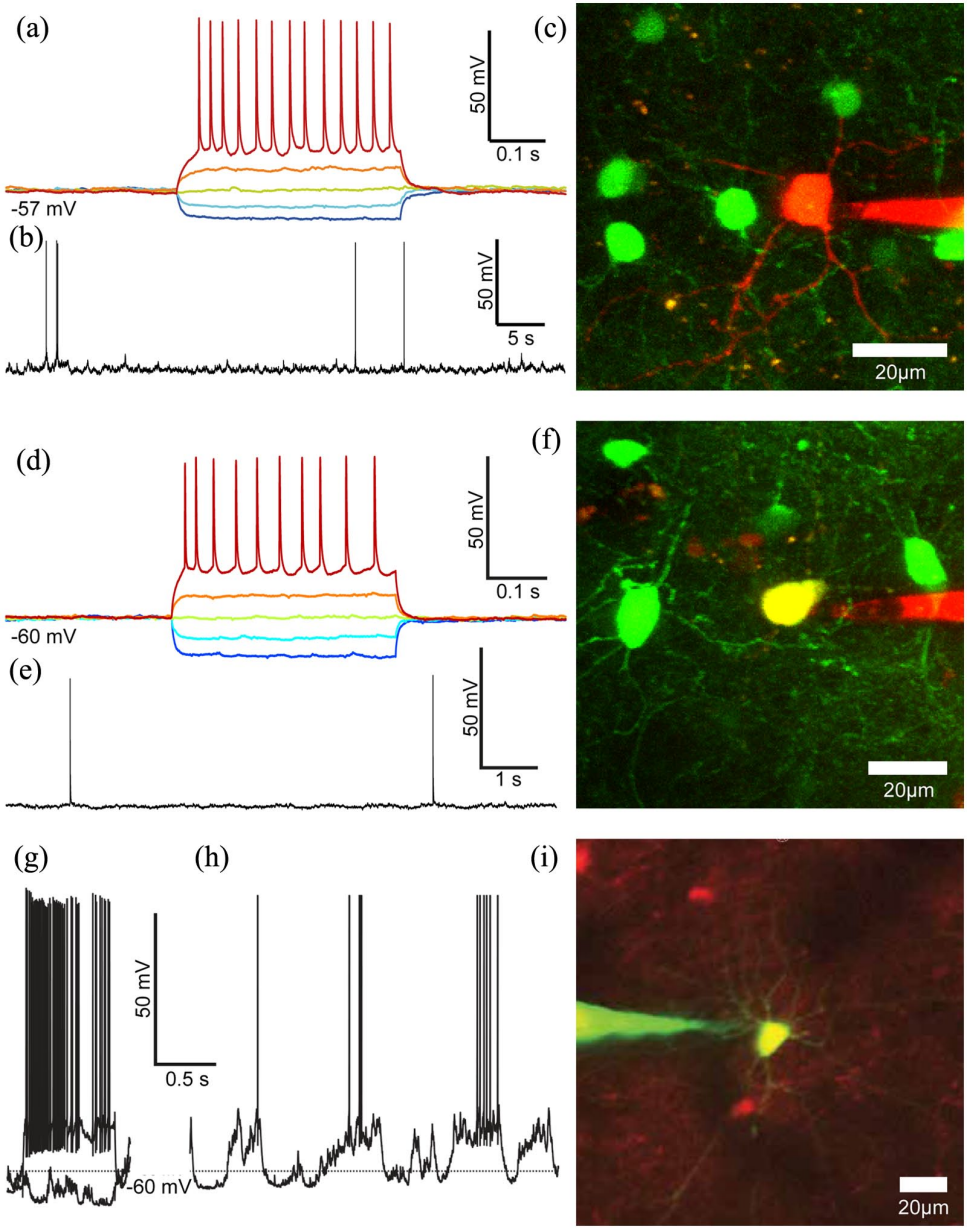
Examples of robotic two-photon targeted whole-cell recordings in vivo. (a) Current-clamp traces during current injection (400-ms-long pulses from −100 to +100 pA in 50-pA steps) and (b) at rest for a robotically patched GFP-positive neuron in the neocortex of a GAD67-GFP mouse. (c) Maximum intensity *z*-projection of a two-photon stack image of the patched neocortical interneuron (recordings in a and b) and the electrode, acquired after the recording (adapted from Annecchino et al. (2017)). (d) Current-clamp traces during current injection (400-ms-long pulses from −100 to +150 pA in 50-pA steps) and (e) at rest for a patched neuron in the neocortex of a GAD67-GFP (depth – 142 µm from the brain surface). (f) Maximum intensity *z*-projection of a two-photon stack image of the patched neocortical interneuron (recordings in d and e) and the electrode, acquired after the recording (adapted from Annecchino et al. (2017)). (g) Current-clamp traces during current injection (400-ms-long pulses from −100 and +200 pA) and (h) at rest for a robotically patched GFP-positive neuron in the neocortex of a PV-Cre × Ai14 mouse. (i) Maximum intensity *z*-projection of a two-photon stack image a patched neocortical interneuron and the electrode, acquired after the recording (Suk et al. (2017)).

The system presented by Annecchino et al. (2017) was tested on parvalbumin (PV)-positive interneurons (Figure 6(a)–(f)) in the neocortex and Purkinje cells in the cerebellum of GAD67-GFP transgenic mouse, obtaining a seal 46.6% of the times, while a successful WCR was obtained on 22.2% of occasions. A limited number of tests were also performed on pyramidal neurons and astrocytes in the neocortex of wild-type mice bulk loaded with AM dyes and/or sulforhodamine. Similar values were also obtained by the implementation of Suk et al. (2017) also targeting PV-positive interneurons (Figure 6(g) and (h)) in the neocortex of a PV-Cre × Ai14 mouse, achieving seals 38.9% and WCR 29.2% of times, respectively. Suk et al. (2017) also tested targeting with CaMKIIa-positive neurons, obtaining similar results (29.9% for a seal and 20.0% for a WCR). The time required for patching by both systems is comparable to a human operator performing classic manual two-photon targeted WCR in vivo (the system implemented by Annecchino et al. achieved a WCR in 6.1 ± 0.6 min while the implementation reported in Suk et al. 10 ± 3 min). Under optimal conditions (based on good cellular fluorescent labelling, no cortical bleeding, and low heartbeat and respiration movement), the duration and quality of the recordings were comparable to those of cortical layer 2/3 pyramidal neurons obtained by blind in vivo WCR in the mouse cortex (Annecchino et al., 2017; DeWeese, 2007; Kodandaramaiah et al., 2012, 2018; Margrie et al., 2002; Suk et al., 2017).

Although independently developed, many of the design and methodological details in the two systems appear similar. Both platforms use similar pipette internal pressure ranges (although using different devices and control method), inter-leave target position assessment with trajectory correction and approaching step, calculate the optimal entry point by assessing the shortest axial trajectory to the target cell, and during the initial penetration step approach the target cell axially. Apart from the different programming languages used for their development (LabView vs MATLAB), another difference can be identified in the pipette navigation strategy used during the final approach to the target cell. The approach taken by Suk et al. (2017) (when the pipette tip is within 50 µm from the initial target cell location) was to (1) find the target cell’s centre of mass, (2) reposition the pipette horizontally (in the *x–y* plane) directly above cell centre of mass, and (3) making the final approach to the target cell by taking 3-µm vertical steps (in the *z* direction) towards the target cell (Figure 5(f) and (g)). However, Annecchino et al., proceed by (1) monitoring the target cell centre of mass position, (2) repositioning the pipette horizontally (in the *x–y* plane) at the intersection point with the axial trajectory parallel to the pipette and passing through the cell centre of mass, (3) approaching the target cell by executing a purely axial penetration step (Figure 5(c)), therefore minimising lateral or vertical movements near to the target cell and thus tissue damage.

## Discussion

Since their inception, automatic patch clamp platforms have evolved considerably, demonstrated capabilities of addressing both voltage- and ligand-gated channels and have been showing the potential to play a pivotal role in drug discovery and biomedical research (for detailed reviews see Wang and Li (2003), Dunlop et al. (2008), Stoelzle et al. (2011), Obergrussberger et al. (2015), Rajamohan et al. (2016), Fang (2017), Obergrussberger et al. (2017), Bell and Dallas (2017), and Obergrussberger et al. (2018)). Ion channels play a fundamental role in regulating multiple aspect of neuronal physiology such as resting membrane potential, AP propagation, transmitter release, synaptic transmission, and plasticity. Ion channels also constitute the main therapeutic targets for a plethora of neurodegenerative or neuropathic conditions, ranging from cognitive health and cardiovascular diseases to chronic pain (Conte Camerino et al., 2007; Humphries and Dart, 2015; Markman and Dworkin, 2006; Tibbs et al., 2016; Zamponi, 2016). As an example, pain is a worldwide health concern with a significant unmet need for improved treatments (Ji et al., 2013, 2014, 2016). Research into treatment of this complex condition has substantial potential to benefit from automated patch clamp technology. The fundamental role of a particular type of a type of voltage-gated sodium channel, Na_*v*_1.7, in nociception or pain perception has been confirmed in several studies, attracting the attention of the scientific and clinical community working on a broader spectrum of conditions associated with pain (Cox et al., 2006; Lee et al., 2014; Minett et al., 2015).

Automated platforms have already been used and compared to manual approaches (Dunlop et al., 2008; Haraguchi et al., 2015; Li et al., 2017; Milligan et al., 2009; Stoelzle et al., 2011; Yu et al., 2016) in the context of Na_*v*_1.7 mutation characterisation (Chambers et al., 2016; Estacion et al., 2010), confirming the sodium ramp current enhancement (Dib-Hajj et al., 2007), and suggesting roles in dorsal root ganglion (DRG) neurons hyperexcitability conditions and chronic pain (Sheets et al., 2007). Several other instances of automated patch clamp paradigms involving the study of Na_*v*_1.7 channel have also confirmed the important roles played by this particular sodium channel (Alexandrou et al., 2016; Deuis et al., 2017; Klint et al., 2015). Other promising molecular pain targets that have been examiner with automated patching platforms are the glycine receptor (Gilbert et al., 2009), hyperpolarization-activated cation nonselective (HCN) channels channels (Vasilyev et al., 2009), Ca_*v*_3 calcium channels (Swensen et al., 2012; Xie et al., 2007; Zamponi, 2016), and GABA_*A*_ and GABA_*B*_ receptors (Enna and McCarson, 2006; Ji and Neugebauer, 2011; Kahle et al., 2016; Knabl et al., 2008) (see also Bagal et al. (2014) and Chambard et al. (2014) for reviews).

The introduction of novel automatic patch clamp devices can be an important driving force and play a key role in moving safety pharmacology assessment to an earlier stage of the drug discovery process. For example, the Cellaxess Elektra platform allows transfection via siRNA and cDNA of primary adherent cell types in genomic screening applications, as well as delivery of small molecules and antibodies in lead identification and target validation. This system is at the core of a joint effort of Cellectricon, Pfizer, and Censo Biotechnology, which using human induced pluripotent stem cell-derived neurons, aims to develop a humanised drug discovery platform to target chronic pain (Tams et al., 2017; Whiteside et al., 2013; Young et al., 2014). Thorough validation of the platform has not yet been reported. Although the benefits of automation for research and drug discovery are evident, there are still several instances of mammalian cell-based assays that automated platforms struggle to handle (Dunlop et al., 2008; Franz et al., 2017). Further development is still required to optimise conditions for both cell performance and assay reproducibility.

Cell biology, electrophysiology, live imaging, and postmortem histological assays can benefit immensely from robotic automation and inform the implementation of novel technologies with the potential to be deployable in the clinical setting. In vivo experimental neuroscience seems particularly amenable to robotic automation, as demonstrated in multiple instances of fully automated blind (Annecchino et al., 2017; Desai et al., 2015; Kodandaramaiah et al., 2012, 2018) and visually guided (Annecchino et al., 2017; Suk et al., 2017) whole-cell patch clamp recordings. Automated targeted electrophysiological interrogation of specific cells has the potential to provide not only readout of the intrinsic excitability but also a direct means to determine the responsiveness of specific subtypes of neurons to different stimuli, with high sampling throughput in comparison to sampling cells with ‘blind’ approaches (which must sample a very large number of cells in order to obtain a useful number of a any but the most numerous cell types). In future generations, such platforms could increase throughput and also enable multi-cell recordings in vivo (Jouhanneau et al., 2015; Pala and Petersen, 2015) allowing high-resolution patch clamp interrogation of individual cells at multiple sites in communicating networks. Novel applications combining robotic targeted WCR system with two-photon functional imaging (Schuck et al., 2014, 2015, 2018; Schultz et al., 2017), together with advances in automated analysis of two-photon imaging data (Giovannucci et al., 2017; Onativia et al., 2013; Reynolds et al., 2017), may extend our ability to study network-related phenomena and potentially forge a direct link between system and cellular neuroscience.

The increasing availability of genetically modified animals showing selective labelling in subpopulations of cells opens up numerous potential applications for robotic systems, in particular for elucidating the role of the diverse and complex set of individual cell types that make up the cortical circuit. Automated patch clamp physiology is also applicable to mouse models of diseases; however, this may involve incorporation of fluorescent proteins into the transgenic disease model itself, or simply injecting a virus targeting a particular cell class into the desired brain area. Another important application for robotic visually guided whole-cell electrophysiology that is likely to receive further attention is assessment of the electrophysiological responses of individual cells transfected (and labelled) by recombinant viruses, plasmid DNA, or peptides suspected to modulate physiological functions. Whole-cell patch clamp can be used also for extraction of cytosolic contents for transcriptomic single-cell analysis (Eberwine et al., 1992). On particular, single-cell RNA-sequencing (scRNA-seq) method, known as Patch-seq, seems to be particularly promising as it makes information about the anatomical position, morphology, connectivity, electrophysiological properties of the cell within the local circuit simultaneously accessible in both tissue slices (Cadwell et al., 2016; Fuzik et al., 2016) and in in vivo preparation (Cadwell et al., 2016). Single-cell RNA expression analysis provides an unbiased strategy to characterise and classify (rare) cell types and may motivate the further development of visually guided methods combining electrophysiological recordings and phenotypical screening with high sampling throughput, in comparison to sampling cells with ‘blind’ approaches (Qiu et al., 2012). Automated two-photon targeted scRNA-seq has the potential to yield more biologically complex information, and be especially useful in reverse engineering the molecular and cellular mechanisms of single-cell and network neuronal computation, while capturing important aspects of human disease mechanisms leading to phenotypic changes and possible therapeutic developments.

Robotic automation of whole-cell electrophysiology has achieved astonishing results, but its broad implementation in custom workflows and integration with existing laboratory hardware can still be challenging, especially in complex in vivo experimental paradigms. The impact of the human factor on the end results of surgical (craniotomies and durotomies) and electrode preparation (pipette pulling and internal solution making) is still a critical factor, often reducing the yield and delaying experimental success. When considering usability issues, modularity requirements, and prospective throughput improvements enabling large arrays of recording electrodes to be operated in parallel, the need for more advanced robotic control strategies, novel microfabrication techniques, and improved electrode materials becomes even more compelling. Further miniaturisation of micropipettes (and the hardware used for their control), advances in robotic surgery (Pak et al., 2015), pipette fabrication (Pak et al., 2011), tip cleaning for reuse (Kolb et al., 2016), improved computer navigation control (Stoy et al., 2017), and automated electrode replacement between trials can dramatically improve the density and throughput of paradigms for electrophysiological interrogation and phenotypical screening of multiple targeted elements of neuronal circuit.

The human nervous system is perhaps the most complex organ in the body and, unfortunately, remains particularly difficult to manipulate and study. This creates specific sets of challenges when studying neurodegenerative diseases and neuropathies. Automated patch clamp platforms have the potential to enable the high-throughput and high-content study of the neural correlates of neurological diseases in vivo to identify anatomical, physiological, and pharmacological pathways and help elucidate the specific disease initiation and progression. As well as enabling hitherto impracticable questions to be addressed concerning neural circuit function, this may be of great utility in developing and testing pharmaceutical and gene therapeutic approaches to treating nervous system disorders.
